# Germline *PRKACA* amplification-associated primary pigmented nodular adrenocortical disease: a case report and literature review

**DOI:** 10.20945/2359-4292-2022-0491

**Published:** 2023-12-01

**Authors:** Wang-Rong Yang, Xing-Huan Liang, Ying-Fen Qin, Hai-Yan Yang, Shu-Zhan He, Zhen-Xing Huang, Yu-Ping Liu, Zuo-Jie Luo

**Affiliations:** 1 First Affiliated Hospital of Guangxi Medical University Department of Endocrinology Nanning China Department of Endocrinology, the First Affiliated Hospital of Guangxi Medical University, Nanning, China; 2 Ruikang Hospital of Guangxi University of Chinese Medicine Department of Endocrinology Nanning China Department of Endocrinology, the Ruikang Hospital of Guangxi University of Chinese Medicine, Nanning, China

## Abstract

Primary pigmented nodular adrenocortical disease (PPNAD) is a rare adrenocorticotropin hormone (ACTH)-independent Cushing's syndrome (CS). Pediatric patients with PPNAD typically have unusual skin lesions and slow growth with unknown causes. We present a case of a female Chinese patient with PPNAD caused by the germline *PRKACA* gene copy number gain of chromosome 19. The patient initially presented with kidney stones, short stature, and obesity. After further testing, it was discovered that the patient had diabetes, mild hypertension, low bone mass, a low ACTH level, and hypercortisolemia, and neither the low-dose or high-dose dexamethasone suppression test was able to inhibit hematuric cortisol, which paradoxically increased. PPNAD was pathologically diagnosed after unilateral adrenalectomy. Chromosome microarrays and whole exon sequencing analyses of the peripheral blood, as well as testing of sectioned adrenal tissue, showed a rise in the copy number of the duplication-containing *PRKACA* gene on chromosome 19p13.13p13.12, a de novo but not heritable gene defect that causes disease. The clinical signs and symptoms supported the diagnosis of Carney complex (CNC). One significant mechanism of CNC pathogenesis may be the rise in germline *PRKACA* copy number of chromosome 19. When assessing PPNAD patients for CNC, the possibility of *PRKACA* gene amplification should be considered. The effect of *PRKACA* gene amplification on the clinical manifestations of CNC needs to be confirmed by more cases.

## INTRODUCTION

Multiple clinical symptoms connected to endogenous cortisol overproduction make up the clinical syndrome known as Cushing's syndrome (CS), which is also characterized by high rates of morbidity and mortality ( 1 ). Clinically, CS can be categorized as ACTH-dependent and ACTH-independent. Bilateral adrenal hyperplasia (BAH), cortisol-producing adenomas (CPA), and adrenal cortical cancer (ACC) are causes of ACTH-independent CS. One of the most crucial pathogenic mechanisms of BAH and CPA is abnormal cAMP-PKA signalling, which is linked to pathogenic variants in several genes, including *GNAS, PRKAR1A, PRKACA, PRKACB* , *PDE11A* , and *PDE8B* ( 2 – 5 ). Pathogenic somatic *PRKACA* mutation and germline *PRKACA* copy number gain are associated with CPA and BAH, respectively.

PPNAD is a type of BAH. Histologically, it is characterized by micronodules with unclear boundaries, atrophic internodal tissues, and rich lipofuscin in the capsule of intracellular nodules ( 6 ). The adrenal gland may display a range of histological types in CS patients with germline *PRKACA* amplification ( 7 ).

Patients with PPNAD should be evaluated for diagnosis of Carney complex (CNC) as it is a common adrenal symptom of the disease. CNC is mainly associated with the inactivation mutation of germline *PRKAR1A* . Herein we report a rare new case of PPNAD with facial lentigines, and neither low-dose dexamethasone suppression test (LDDST) nor high-dose dexamethasone suppression test (HDDST) was able to inhibit urine free cortisol (UFC), which paradoxically increased. Notably, genetic testing showed germline *PRKACA* amplification. Based on the findings for the present case, CNC was diagnosed, and we consider that its occurrence was related to the germline *PRKACA* amplification.

## CASE PRESENTATION

A 22-year-old woman with right-side low back pain was hospitalized in our institution's Department of Urology. When she was 8 years old, right-side low back pain, which had not previously been treated as it was a minor symptom, led to a diagnosis of right-side kidney stones. She visited the clinic when she was 16 years old for evaluation for the potential of short stature. After routine examinations, she was informed that no abnormality was found. She was then referred to the Department of Endocrinology for further examinations when abnormal hyperglycemia was detected during the present hospitalization in the Department of Urology. Based on her detailed medical history, the patient had begun to be obese at the age of 8 years. Then lentigines appeared on her face when she was 10 years old, and she experienced menarche at the age of 14 years. She was the second child of the family. Her parents were healthy, and no other children in the family had similar symptoms. The body heights of her family members were: father, 161 cm; mother, 154 cm; younger sister, 160 cm; older sister, 154 cm; and younger brother, 178 cm.

### Physical examination

**Figure 1:**
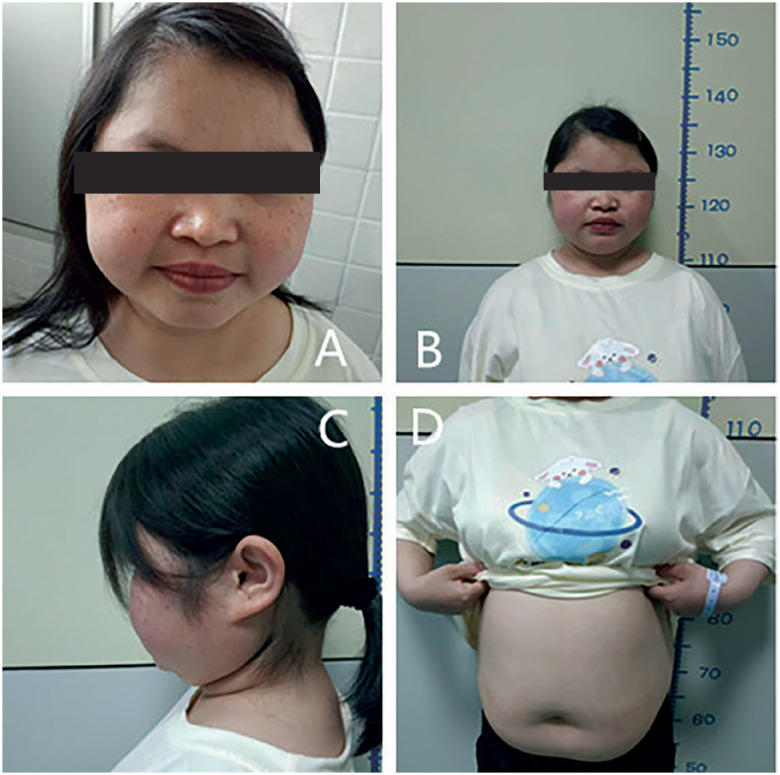
Signs of Cushing's syndrome on physical examination of the patient. **A** : signs of full-moon face and lentigines on her face with acne; **B** : signs of full-moon face with stature short than her target height; **C** : fat pads on the shoulder and back; and **D** : no typical wide purple striae on the abdomen.

The blood pressure of the patient was 148/118 mmHg at admission. Her body weight and height were 50 kg and 137 cm, respectively. Her abdominal circumference was 92 cm, and her hip circumference was 81.5 cm. By observing the appearance of the patient, snowflake-like dandruff could be seen on her scalp, and she had many lentigines on her face with acne. Typical signs of full-moon face, centripetal obesity, and fat pads on the shoulder and back were also observed, with slightly thinned skin over her whole body. No typical wide purple striae were detected on the abdomen ( Figure 1 ). Her breast development was normal, while the development of the labium minus was poor. She reported obvious percussion pain in the right kidney area.

### Laboratory findings

Table 1 shows the results of the oral glucose tolerance test (OGTT) for the patient. A measurement of the rhythm of serum cortisol showed levels of 205.0 ng/mL at 8 AM, 199.0 ng/mL at 4 PM, and 203.0 ng/mL at 0 AM. The serum and urinary cortisol levels were found to be significantly increased. The diurnal rhythm of cortisol was absent, and the plasma ACTH level was markedly low.

**Table 1:** Results of oral glucose tolerance test

	0 h	1 h	2 h	3 h
Blood glucose (3.9-6.1 mmoL/L)	6.37	19.67	20.93	12.86
C-peptide (0.3-3.73 ng/mL)	3.88	11.50	20.4	20.00

The results of low-dose and high-dose dexamethasone suppression tests are presented in Table 2 . The patient's serum cortisol level was increased abnormally after the overnight low-dose dexamethasone suppression test (LDDST, 0.75 mg every 8 hours for 48 hours) and after the overnight high-dose dexamethasone suppression test (HDDST, 2 mg every 6 hours for 48 hours). The patient's serum cortisol level exceeded the baseline values by 37.3% and 66.7% after LDDST and HDDST, respectively. LDDST and HDDST revealed that the patient's UFC level was not inhibited, and the UFC level after HDDST was higher than that after LDDST, indicating a paradoxical increase ( Table 2 ).

**Table 2:** Results of dynamic testing of serum and urinary cortisol and serum ACTH

	24-h urine volume (mL)	24-h urinary cortisol (3.5-45 µg/24 h)	ACTH (6.0-48 pg/mL)	Serum cortisol at 8 AM (57.2-194.2 ng/mL)
Baseline	2,500	116.3	4.0	132.0
1 mg LDDST			3.4	126.0
2 mg LDDST	1,700	47.1	3.0	181.2
8 mg HDDST	1,600	83.7	2.8	220.0

LDDST: low-dose dexamethasone suppression test; HDDST: high-dose dexamethasone suppression test; ACTH: adrenocorticotropin hormone.

### Imaging findings

**Figure 2:**
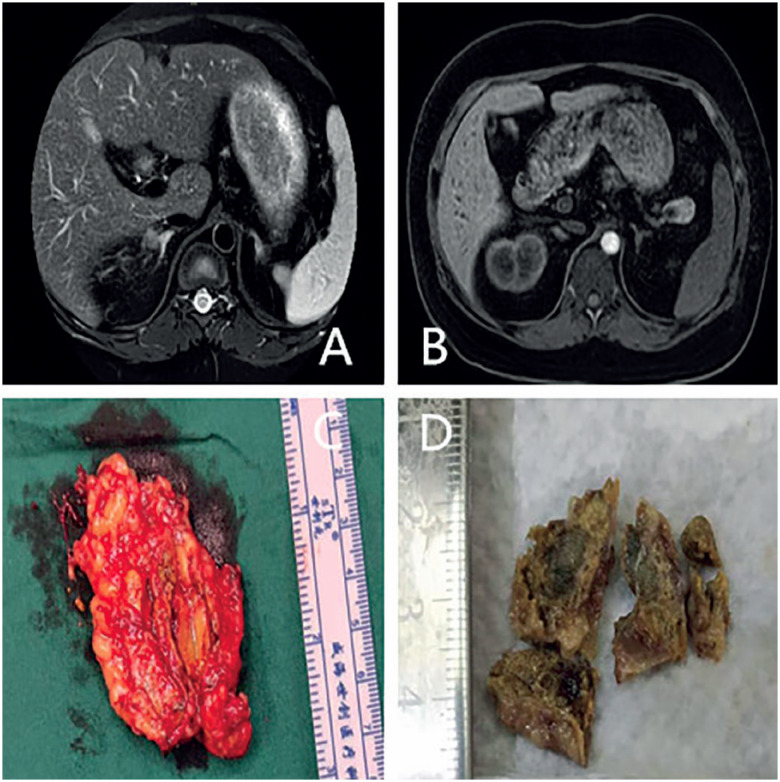
Adrenal MRI findings and gross tissue examination of the resected adrenal gland. **A:** adrenal MRI showed small nodules in the right slightly thickened adrenal gland; **B** : adrenal MRI showed no abnormal nodule in the left slightly thickened adrenal gland; **C** and **D** : gross tissue examination of the resected adrenal gland revealed golden yellow and brown nodular hyperplasia of the adrenal gland.

MRI showed that the bilateral adrenal glands were slightly thickened, with nodular thickening at some locations, measuring approximately 10 mm in diameter ( Figures 2A and 2B ). Echocardiography excluded atrial myxoma. Thyroid ultrasound showed multiple echoless nodules in the bilateral thyroid measuring about 0.2 × 0.2 cm in size, indicating small cystic nodules on both sides of the thyroid (TI-RADS type 2). A pituitary MRI exhibited no tumor in the pituitary tissue. Lumbar spine X-ray examination revealed low bone mass.

### Surgical treatment and pathological findings

**Figure 3:**
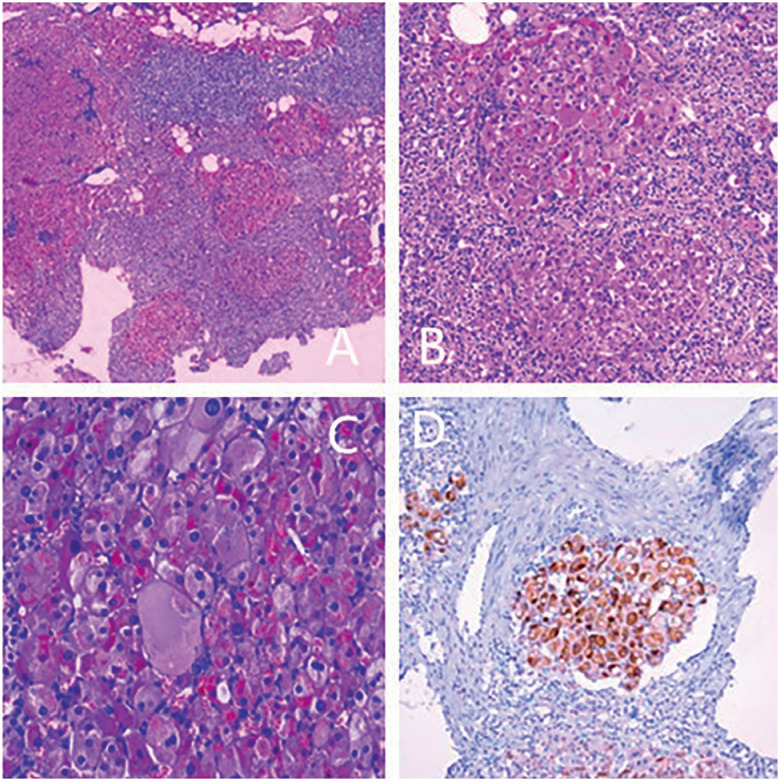
Pathological findings of the resected left adrenal gland tissue; **A:** a 4×10 HE staining image shows multiple small nodule hyperplasia and intertubercular cortical atrophy of the adrenal gland tissue; **B:** a 10×10 HE staining image shows lipofuscin in small nodules of the adrenal gland tissue; **C:** a 40×10 HE staining image shows accumulation of eosinophil granules in the cytoplasm with lipofuscin in the cells of the adrenal gland tissue; and **D** : a 10×10 immunohistochemistry staining image shows diffuse α-Inhibin expression in the cells of the hypertrophic nodule of the upper gland cortex, but not in cells of the peripheral atrophic cortical of the adrenal gland.

The patient was treated with left unilateral adrenalectomy. During the operation, scattered nodular changes were observed on the left adrenal surface. The nodules were golden yellow and dark brown, with a diameter of 0.2∼1.0 cm ( Figures 2C and 2D ). Pathological examination (hematoxylin and eosin [HE] staining) revealed multiple round or oval nodules in the cortex of the left adrenal gland ( Figure 3A-C ), along with the accumulation of eosinophil and brown pigmentation. The cells of the adrenal glands were closely arranged in the pattern of nests and beams. The border of the nodules was unclear without a capsule, and the cortex between the nodules was atrophic. Immunohistochemical staining showed that α-Inhibin is diffusely expressed in the cells of the hypertrophic nodule of the upper gland cortex, but not in cells of the peripheral atrophic cortical of the adrenal gland ( Figure 3D ). Additionally, the cells of the resected adrenal gland stained positively for synaptophysin (Syn), S100, and glycogen, but negatively for chromogranin A (CgA), HMB-45, and iron. Accordingly, a pathological diagnosis of PPNAD was confirmed.

### Genomic DNA extraction, high-throughput DNA sequencing, gene chip validation, and qPCR validation

After obtaining peripheral venous blood samples from the patient and her parents (2 mL each), the genomic DNA was extracted with the QIAGEN DNA Extraction Kit (QIAGEN, Germany).

**Figure 4:**
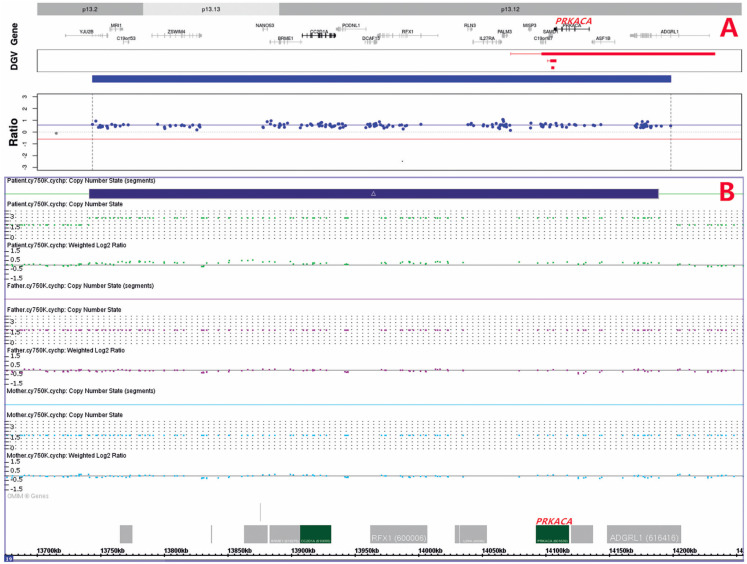
Identification of the genomic copy number gain involving the key gene *PRKACA* of the patient. A, duplication in the 19p13.2-p13.12 region identified by the bioinformatics analysis of whole exon sequencing data (Log2 Ratio = 0.56); and B, duplication in the 19p13.13p13.12 region validated by the Affymetrix CytoScan 750 K array, where duplications were excluded in her parents.

The extracted DNA was segmented using DNA enzyme and purified by the magnetic bead method before being amplified by PCR and connected with the connector sequence. The target region of the whole exon group was captured and purified using the IDT xGen Exome Research panel probe, and the final library was sequenced on a NovaSeq 6000 sequencer (Illumina, USA). The depth of the captured sequence was calculated via ExomeDepth and the internal algorithm ( 8 ), and subsequent copy number variation (CNV) analysis was also performed. The sequencing results indicated an ∼426-kb copy number gain in the region of chromosomal chr19p13.2 p13.12 (GRCh37: chr19: 13862572-14288606) in genomic DNA. This genomic region of copy number gain variation contained the key gene *PRKACA* ( Figure 4A ). Whole exon sequencing analyses were not performed for the parents of the patient.

Subsequently, the CytoScan HD (1950000 CNV probes+750000 SNP probes) gene chip (Affymetrix, USA) was used to analyze the samples. The raw data obtained were analyzed with the Affymetrix Chromosome Analysis Suite Software. The results of the CNV analysis were interpreted according to the international genome CNV polymorphism database including the DGV, UCSC, Genome browser, DECIPHER, and ISCA databases, as well as the previous relevant literature ( 9 ). The results of the microarray analysis confirmed the copy number gain located in arr [GRCh38] 19p13.13p13.12 (13740503-14187904) x3 of the patient, indicating the existence of duplication, which included the key gene *PRKACA* ( Figure 4B ). No such changes were observed in the parents of the patient. The results of adrenal tissue examination (formalin fixed and paraffin embedded sections), which also revealed increased *PRKACA* gene copy number, were consistent with the peripheral blood test.

**Figure 5:**
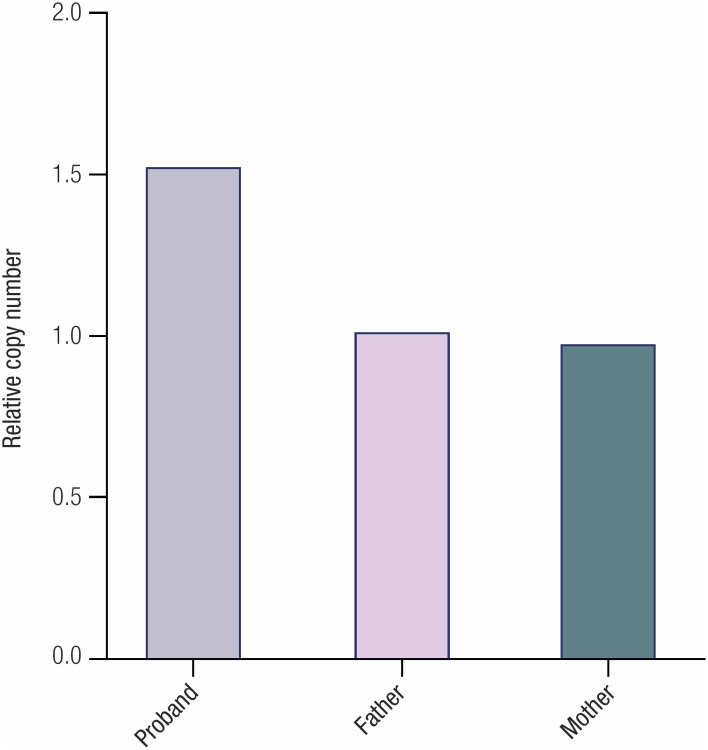
qPCR analysis of genomic DNA copies of the *PRKACA* gene in the patient and her parents.

Finally, a quantitative real-time PCR (qPCR) was performed to validate the genomic DNA copies of the *PRKACA* gene. The results showed that the number of DNA copies of the *PRKACA* gene was markedly higher in the patient but not in the patient's parents ( Figure 5 ).

PPNAD caused by the germline *PRKACA* copy number gain has been reported in the human genetic database, suggesting that the genetic pathogenic alteration is de novo but not inherited.

The clinical diagnosis for the present case was CNC ( Table 3 ).

**Table 3:** CNC-related clinical findings in the reported case

Item	Clinical findings	Current case
Skin	Lentigines (mucosa of lip, conjunctiva, inner canthus, outer canthus, perineum) Blue nevus	Yes
Pituitary	Somatomammotroph hyperplasia Asymptomatic elevation of GH, IGF-1 or prolactin GH-producing adenoma with acromegaly	No
Thyroid	Thyroid cancer or multiple hypoechoic nodules on prepubertal ultrasound	No
Adrenal gland	PPNAD, dexamethasone suppression test increased abnormally	Yes
Gonad	Large-cell calcifying Sertoli cell tumor of testis or specific calcification foci on ultrasound Ovarian cysts	No
Others	Osteochondromyxoma Psammomatous melanotic schwannoma Multiple ductal adenomas of breast Myxoma (skin, mucosa, heart)	No
Gene	Activating mutation and copy number variation of *PRKACA* and *PRKACB* Inactivating mutation of *PRKARlA*	Yes

### Follow-up visit

Measurement of the rhythm of serum cortisol revealed cortisol levels of 484, 523, and 498 ng/mL at 8 AM, 4 PM, and 0 AM, respectively, on the third day after surgery. The patient's serum cortisol level at 8 AM 3 months after the operation was normal at the neighborhood hospital. The patient's self-measured blood glucose dramatically improved, likely due to factors connected to the patient (menstruation), which also prevented the detection of UFC and the cortisol rhythm. During this period, she was infected with the novel coronavirus and developed a fever, but she showed no symptoms of adrenal cortical dysfunction such as nausea, vomiting, fatigue, etc. The patient is still being followed up now.

## DISCUSSION AND CONCLUSIONS

In this patient, the initial manifestation of the disease was kidney stones, which occurred as early as 8 years of age. Short stature and obesity were also noticed during her puberty. However, CS was not diagnosed in this patient until the currently described admission, because the patient did not have typical purple striae and easy bruising of skin during physical examination or an abnormal serum cortisol level during previous hospitalizations. Considering that hyperglycemia, low bone mass, and short stature reflect the continuous effects of long-term hypercortisolemia, the possibility of cyclical Cushing syndrome (CCS) cannot be ruled out.

PPNAD is usually cyclical or intermittent CS (namely CCS) ( 10 , 11 ). CCS is characterized by repeated episodes of excessive cortisol production (relapse) alternate with intervals of normal cortisol production (remission). The so-called hypercortisolism cycle contains weekly phases that can last anywhere from a few days to several years and can recur regularly or erratically. There have been reports of cycles lasting anywhere from 12 hours to 85 days ( 12 , 13 ). Ninety-five percent of CCS patients were reported to have two or more of the typical signs of CS, such as hypertension, central obesity, a buffalo back, a full moon face, puffiness, or purple lines. Acne, hirsutism, amenorrhea, altered glucose tolerance, and abnormal mood are some other concomitant clinical signs ( 14 ). However, the likelihood of something being missed during the clinical diagnosis process is very high if the patient exhibits fewer typical CS symptoms or they are not seen in the endocrinology department. The presence of three peaks and two troughs in cortisol synthesis can be regarded as periodic to support a definitive diagnosis of CCS ( 15 , 16 ). However, the diagnostic procedure is also challenging.

Weight gain with decreasing growth velocity is the most typical symptom in children with CS ( 17 , 18 ). Short stature has been seen in 29% of PPNAD patients and may result from the side effects of hypercortisolemia. Growth hormone and insulin-like growth factor-1 synthesis may be inhibited by persistently high cortisol levels ( 19 , 20 ).

Through the growth hormone (GH)-insulin-like growth factor (IGF-1) axis, bone cells, vitamin D and calcium metabolism, sex hormones, PTH, and other mechanisms, excessive glucocorticoids have an impact on BMD. GH and IGF-1 are crucial regulators in maintaining the stability of the bone environment as well as significant factors in determining longitudinal bone development and bone gain ( 21 ). GH can indirectly boost osteoblast activity by promoting IGF-1 production and release in addition to directly stimulating the proliferation and differentiation of osteoblasts. The GH/IGF-1 axis can be severely inhibited by glucocorticoids at super-physiological doses, which can negatively impact bone density. Increased levels of glucocorticoids directly limit osteoblast proliferation and differentiation, which lowers bone formation and increases osteoclast activity and bone resorption ( 22 ). By blocking the effects of vitamin D, glucocorticoids limit the expression of calcium channels in the duodenum, which results in secondary hyperparathyroidism and lowers calcium absorption in the intestine ( 23 ). PPNAD's negative feedback lowers ACTH levels, which reduces sex hormone synthesis and secretion as well as impairs bone metabolism and eventually results in osteopenia ( 24 ).

Imaging diagnosis of PPNAD is difficult. Histologically, according to a previous report ( 25 ), PPNAD is characterized by micronodules, and approximately half of patients with PPNAD present with no abnormal adrenal morphology. Clinically, PPNAD occurs as a sporadic condition in approximately 33% of cases and as a familial condition and part of CNC in 66% of cases. PPNAD is linked to the pathogenic variant of multiple genes, and abnormal cAMP-PKA signaling is a key factor in its process.

Increases in *PRKACA* copy number associated with somatic variation, germline variation and mosaic variation have been reported to be involved in CS ( 26 , 27 ). The adrenal pathologic phenotypes of CS cases with germline *PRKACA* copy number gain are not identical and include PPNAD, isolated adrenocortical microsarcoidosis (iMAD), primary hyperadrenocortical hyperplasia of the macronodular adrenocortical hyperplasia (PMAH), and iMAD/PMAH combination ( 3 , 28 ) ( Table 4 ). This may be related to the degree of *PRKACA* amplification. Therefore, different genetic changes may lead to the same adrenal pathological phenotype in this scenario, while the same genetic changes may also lead to different adrenal pathological phenotypes. Currently, though somatic *PRKACA* mutation and germline *PRKACA* copy number gain are associated with APC and BAH, respectively, the correlations of specific genetic changes in *PRKACA* mutations and the clinical phenotype remain unclear, and the underlying mechanisms remain to be investigated in the future. Through our review of the Chinese and English literature, we identified 15 different types of *PRKACA* mutations and copy number gain in 17 patients, as well as the corresponding histopathological types and clinical phenotypes (18 cases including the present case) from 9 previous reports ( 2 , 7 , 28 – 34 ) ( Table 4 ). The clinical phenotypes of these cases vary significantly, including ACTH-independent CS, CS, primary aldosteronism, etc. These findings suggest a complicated interaction between genetic alterations and pathological phenotypes that should be investigated in future studies ( 7 ).

**Table 4:** Clinical diagnosis and genetic defects of *PRKACA* in current and previously reported cases

No	Sex	Age (years)	Pathologic diagnosis	Clinical diagnosis	PRKACA defects	Change in amino acid
1 Current case	Female	22	PPNAD	PPNAD	19p13.13p13.12 genomic region with copy number gain variation contained the key gene *PRKACA*	NR
2 (28)	Male	8	BAH	CS	2.7-Mb duplication encompassing *PRKACA* on chromosome 19p13.2p13.12, DUP-NML-DUP	NR
3 (28)	Male	2.6	iMAD	CS	294-kb gain at chromosome 19p13.13p13.12, DUP-Trp, *PRKACA* was included in triplicated segment	NR
4 (28)	Male	2.7	iMAD/PMAH mixed type	CS	551-kb gain on chromosome 19p13.13p13.12, DUP-Trp-DUP, *PRKACA* was included in triplicated segment	NR
5 (28)	Male	39	PMAH	CS	616-kb gain on chromosome 19p13.2	NR
6 (28)	Male	23	PMAH	CS	616-kb gain on chromosome 19p13.2	NR
7 (30)	Female	15	NR	Skeletal syndrome associated with developmental delay	c.858_860GAA	p.K286del
8 (30)	Female	25	PPNAD	ACTH-independent CS	c.899C>T	p.T300M or p.T347M in another isoform
9 (31)	Female	49	NR	CS and PA	c.6T>G	p.L206R
10 (32)	Female	32	NR	PA, adrenal adenomas	c.262C>G	His88Asp
11 (32)	Female	51	NR	PA, adrenal adenomas	c.617A>C	P.Leu206Arg
12 (33)	Female	41	NR	CS, pituitary microadenoma, adrenal macronodule	c.617A>C	P.Leu206Arg
13 (33)	Female	31	NR	CS, pituitary microadenoma, adrenal macronodule	c.639G>T	p.Ser213Arg
14 (34)	NR	NR	NR	ACTH-independent CS, adrenocortical masses	c.600 601ins-GTG	(p.Cys200 Gly201insVal)
15 (34)	NR	NR	NR	ACTH-independent CS, adrenocortical masses	c.639C>G	(p.Ser213Arg)
16 (2)	NR	NR	NR	CS, adrenal adenomas	c.617A→C	p.Leu206Arg
17 (2)	NR	NR	NR	CS, adrenal adenomas	c.595_596insCAC	Leu199_Cys200insTrp
18 (29)	F	21	PPNAD	ACTH-independent CS, PPNAD	c.95A>T	

Note: PA: primary aldosteronism; CS: Cushing's syndrome; BAH: bilateral adrenocortical hyperplasia; iMAD: isolated micronodular adrenocortical disease; PMAH: primary macronodular adrenocortical hyperplasia; NR, not reported.

The multiple neoplasia syndrome known as CNC is either de novo or autosomal dominantly inherited ( 10 ). Myxoma, pigmented schwannoma, and numerous endocrine gland tumors with spotted skin pigmentation are the clinical symptoms ( 11 ). About 75% of cases of PPNAD, a typical adrenal manifestation of CNC, are brought on by the inactivated *PRKAR1A* mutation. On the 2p chromosome, a second location has been found, although the causal gene is still unknown. More genes have recently been found that may be connected to the clinical phenotype of CNC.

A CNC case with germline *PRKACB* amplification was reported in the literature, and the clinical manifestations were acromegaly, pigmented spots, and myxomas ( 35 ). Lodish and cols. reported five cases of BAH caused by germline *PRKACA* amplification with CS ( 28 ). In one case with BAH (but not PPNAD and facial pigmentation), UFC was paradoxically elevated on Liddle's test, CNC was deemed likely. The findings of these previous reports may further expand the diagnostic criteria for CNC ( 11 ).

Given the high incidence of benign thyroid nodules and because the patient's thyroid gland was not examined before puberty, the existence of thyroid nodules was not included in the diagnostic criteria for the present case. The patient's facial pigmentation spots (lentigines), PPNAD and paradoxical positive response of UFC on Liddle's test, and germline *PRKACA* amplification met two main criteria and one secondary criteria, and thus, this case was consistent with the diagnosis of CNC. However, very few similar CNC with germline *PRKACA* amplification have been reported.

Protein kinase A (PKA) regulates several major biological processes, such as metabolism, transcription, cell cycle, and apoptosis ( 36 ). The PKA kinase subunit is activated by germline *PRKACA* amplification and somatic mutations, which also influence cAMP-PKA signaling. It has been reported that patients with triplications action exhibit clinical manifestations at a younger age (around 2 years old), which is consistent with the current case in which clinical manifestations appeared at the age of 8 with a duplication encompassing *PRKACA* ( 28 ). Moreover, the patient's cells showed increased levels of PKA catalytic subunit proteins, suggesting that the gene alteration caused the disease by activating PKA kinase ( 28 ). This finding also raises the possibly that PKA activity has a dose-dependent effect on the phenotype, though more case data are needed to confirm this. Haploinsufficiency of *PRKAR1A* brought on by deficiencies in *PRKAR1A* associated with CNC results in loss of PKA regulatory subunit function, increasing cell proliferation in cAMP-reactive tissues, and tumor growth in CNC-affected tissues due to “unrestricted” catalytic subunit activity ( 11 ). Similar to the *PRKAR1A* abnormality, amplification of the germline *PRKACA* generated the same outcome. This observation provides a possible molecular mechanism for the diagnosis of CNC in the current case. We ultimately identified CNC in this case, which was the first PPNAD case in China to possess germline *PRKACA* amplification.

For the majority patients with PPNAD who have bilateral adrenal lesions, adrenalectomy has become the most effective treatment method ( 37 ). In previous studies, the curative rates for PPNAD with unilateral adrenalectomy, subtotal adrenalectomy, and total adrenalectomy were approximately 30%, 60%, and 90%, respectively ( 38 , 39 ). Following surgery, cortisol levels should be monitored every 6 months to 1 year to evaluate the dynamic changes in serum cortisol levels and to determine whether hormone replacement therapy is needed. However, in our case, resection of unilateral adrenal gland appears to have allowed blood cortisol levels to remain normal, and further monitoring of the patient is necessary.

In conclusion, the germline *PRKACA* amplification in chromosome 19 may play a significant role in the pathogenesis of CNC. When evaluating PPNAD patients for the presence of CNC, the possibility of germline *PRKACA* amplification should be considered. More cases are required to confirm the impact of germline *PRKACA* amplification on the clinical signs and symptoms of CNC.

## Data Availability

the datasets used and/or analyzed during the current study are available from the corresponding author upon reasonable request.
